# LC-MS identification and preparative HPLC isolation of *Frankenia pulverulenta* phenolics with antioxidant and neuroprotective capacities in PC12 cell line

**DOI:** 10.1080/13880209.2016.1278452

**Published:** 2017-02-02

**Authors:** Rim Ben Mansour, Megdiche Ksouri Wided, Stéphanie Cluzet, Stéphanie Krisa, Tristan Richard, Riadh Ksouri

**Affiliations:** a Laboratory of Aromatic and Medicinal Plants, Center of Biotechnology, Technopark of Borj-Cedria (CBBC), Hammam-Lif, Tunisia;; b ISVV, GESVAB EA3675, University of Bordeaux, Villenave d‘Ornon, France

**Keywords:** β-Amyloid peptide, LC-DAD-ESI-MS, fractionation, MTT test, ORAC

## Abstract

**Context:***Frankenia pulverulenta* L. (Frankeniaceae) is a medicinal species with carminative, analgesic and antiviral properties. However, phytochemical investigations, antioxidant and neuroprotective capacities of this plant remain unclear.

**Objective:** This work assesses the phenolic composition of *F. pulverulenta* shoot and root and evaluates their antioxidant and neuroprotective capacities.

**Materials and methods:** Successive fractionation of *F. pulverulenta* shoot and root using 6 solvents were used. Antioxidant capacity of these fractions was assessed through four *in vitro* tests (DPPH, ABTS, Fe-chelating activity and ORAC). Phenolic identification, purification as well as neuroprotective activity of ethyl acetate (EtOAc) fraction and purified molecules were assessed.

**Results:** Among the tested fractions, EtOAc shoot and root fractions possessed considerable phenolic contents (383 and 374 mg GAE/g E, respectively) because of their important ORAC (821 and 1054 mg of TE/g E), DPPH (586 and 750 mg of TE/g) and ABTS (1453 and 1319 mg of TE/g) results. Moreover, gallic acid, quercetin, quercetin galloyl glucoside, trigalloyl hexoside, procyanidin dimers and sulfated flavonoids were identified by LC-DAD-ESI-MS for the first time in this species. The relevant cytoprotective capacity (at 300 μg/mL) against β-amyloid peptide induced toxicity in PC12 cells of EtOAc fractions were corroborated with the chemical composition. In addition, purified molecules were tested for their ORAC and neuroprotective activity. Quercetin showed the best ORAC value (33.55 mmol TE/g polyphenols); nevertheless, procyanidin dimer exhibited an exceptionally efficient neuroprotective activity (100% of viability at 50 μg/mL).

**Discussion and conclusions:** These findings suggest that this halophyte is a promising source of antioxidant and neuroprotective molecules for pharmaceutical purposes.

## Introduction

Nowadays, herbal constituents, especially phenolics, have been received increasing interest owing to their reported beneficial effects on longevity and disease prevention (Lin et al. 2015). The identification of new sources of natural antioxidants is a promising alternative for their use and value in the food industry and in preventive medicine to replace synthetic antioxidants (Tadhani et al. 2007). This is mainly due to their strong biological activity, exceeding those of many synthetic antioxidants that have possible activity as promoters of carcinogenesis (Ksouri et al. 2009). Comprehensive identification of phenolic compounds in food matrices is a crucial starting point for assessing their biological, nutritional and technological properties (Gasperotti et al. 2010). This kind of compounds acts as antioxidants, flavor precursors and as ingredient of our diet, and they have been associated with several health-promoting activities such as decreasing blood sugar levels, reducing body weight, anticarcinogenic, anti-inflammatory, antiaging and antithrombotic capacities (Senevirathne et al. 2006). These activities depend on their structure, in particular the number and positions of the hydroxyl groups and the nature of substitutions on the aromatic rings. Much of the current research interest focus on the study of antioxidant molecules that are able to attenuate the damaging effects of Reactive Oxygen Species (ROS). Excess of ROS in the body can lead to cumulative damage in proteins, lipids and DNA, resulting in the so-called oxidative stress (Yoshikazu & Yuji 2002). Among cellular structure, neurons are particularly vulnerable to ROS, and oxidative stress is one of the main causative factors in the etiology of a number of late onset disorders (Esposito et al. 2006; Tellone et al. 2015). Oxidative stress, which is one of the specific hallmarks of Alzheimer’s disease (AD), promotes neurofibrillary degeneration and death of neurons (Abramov et al. 2004; Lin & Beal 2006). Yatin et al. (1999) reported that the involvement of free radicals in AD includes the presence of elevated levels of protein oxidation, lipid peroxidation products and oxidative damage to mitochondria in AD brains. Furthermore, neurotoxicity of amyloid peptides occurs in conjunction with the presence of oxidative stress associated with the peptide, and excessive accumulation of Aβ peptide in neurons triggers progressive neuronal degeneration (Richard et al. 2011). Implication of Aβ_25–35_ induceing apoptosis in multiple cell types *in vitro* and *in vivo* in human AD brains was suggested by many other reports (Lin & Beal 2006; Bolea et al. 2013; El Bitar et al. 2014; Clarke et al. 2015). Aβ_25–35_ caused a significant decrease in viability with a concomitant increase in apoptosis and morphological abnormalities of nuclei in PC12 cells (Lin & Beal 2006).

There is an international effort to understand the biology of AD to develop primary and secondary prevention strategies, and to develop effective therapeutic interventions for individuals who are already symptomatic (Boada et al. 2014). Hence, the balance between antioxidation and oxidation is believed to be a critical concept for maintaining a healthy biological system (Finkel & Holbrook 2000). Therefore, the search of antioxidants from natural sources has received much attention, and efforts have been made to identify new natural resources for active antioxidant compounds. In Tunisia, a considerable diversity of medicinal halophytes species with multiple interests including therapeutic practices occurs, and many have not been subject to chemical investigations. For example, *Frankenia pulverulenta* L. (Frankeniaceae) is an endemic species from North Africa (Ozenda 1991), growing in the high plateaus and in salt and arid regions of Tunisia. The principal use of this species in local medicine is based on the oral administration of a decoction and gargle prepared from whole plant for its analgesic and carminative properties (Youssef 2013). Phytochemical studies on the genus *Frankenia* and the information on the chemical composition of *F. pulverulenta* are still scarce. Moreover, it was reported that *F. pulverulenta* extract is active against *Herpes simplex* virus type 1 (Sassi et al. 2008). Previous studies focused on the phytochemical identification of flavonoids and phenolic sodium sulfates from *F. thymifolia* Desf. (Harkat et al. 2007), *F. pulverulenta* L. (Harborne 1975), *F. laevis* L. (Hussein 2004) and *F. thymifolia* (Megdiche-Ksouri et al. 2011).

This study describes the optimization of shoot and root fractionation of *F. pulverulenta* using solvents with increasing polarity. This investigation was assessed through different antioxidant activities, such as DPPH, ABTS, metal chelating activity and ORAC tests. In addition, separation and identification of phytochemical composition of EtOAc fractions by LC-DAD-ESI-MS and preparative HPLC as well as the neuroprotective activity on PC12 cell line of acetate fraction and purified molecules were assessed.

## Materials and methods

### Plant material and extraction

*Frankenia pulverulenta* was collected during the vegetative stage in March 2014 from Borj-Cedria (latitude: 36°46′N, longitude: 10°39′E 16 m elevation) at 30 km to Tunis. This halophyte was identified at the Biotechnology Center by Pr Abderrazak Smaoui (CBBC, Technopark of Borj-Cedria), and a voucher specimen [PLM51] was deposited at the Herbarium of the Laboratory of Medicinal and Aromatic Plants at the CBBC. Shoot and roots were air dried. Sample extracts were obtained by magnetic stirring of 150 g of dry matter powder with 1500 mL methanol 80% for 2 h, then the filtrate is evaporated using a rotary evaporator. The obtained filtrate is first extracted with hexane followed by dichloromethane, EtOAc and finally with butanol. The different phases are separated by a separator funnel.

### Total phenolic contents

The total phenolic content (TPC) in aerial parts and roots of extract and fractions were determined by the Folin–Ciocalteu colorimetric method (Singleton & Rossi 1965) adapted to a 96-well plate. Briefly, 100 μL of Folin-Ciocalteu’s reagent was added to 20 μL of extract (1 mg/mL). After 5 min, 80 μL of sodium carbonate (75 g/L) solution were added. After 1 h, the absorbance was measured at 765 nm. The TPC was expressed as mg gallic acid equivalent per g of extract (mg GAE/g E). Experiments were analyzed at least three times and with triplicate samples.

### Radical scavenging assays

Radical scavenging ability against DPPH radical was measured as described by Blois (1958). A volume of 50 μL of each samples (1 mg/mL) were mixed with 150 μL of 200 μM methanolic solution of DPPH in a 96-well plate. The plate was allowed to stand at room temperature in dark for 20 min. The absorbance was measured at 520 nm. Results were expressed as mg of Trolox equivalent per g of extract (mg TE/g).

The scavenging activity of the extracts on ABTS radical cation was estimated according to the method of Re et al. (1999). Briefly, 250 μL of the diluted ABTS^+ ^solution were added to 10 μL of extracts at the concentration of 1 mg/mL (or Trolox). Six min after initial mixing, the absorbance was measured at 734 nm at 30 °C. Results were expressed as mg of Trolox equivalent per g of extract (mg TE/g). All samples were analyzed in triplicate in at least three different experiments.

### Metal chelating activity (MCA)

The chelating activity of the extracts for ferrous ions Fe^+2^ was measured according to the method of Dinis et al. (1994). A volume of 80 μL of deionized water and 40 μL of FeSO_4_ (0.2 mM) were added to the extract (40 μL, 1 mg/mL) and mixed o a 96-well microplate. The reaction was initiated by the addition of 40 μL of ferrozine (2 mM). After 10 min, at room temperature, the absorbance of the Fe^2+^-ferrozine complex was measured at 562 nm. EDTA was used as standard and results were expressed as mg EDTA per gram of extract (mg EDTA/g). All samples were analyzed in triplicate in at least three different experiments.

### ORAC assay

The procedure was modified from the method described by Ou et al. (2001), using Trolox as a control standard. The ORAC assay was carried out in black round-bottom 96-well microplates (Costar) and absorbance was measured with an automated plate reader (Fluostar Optima; BMG Labtech). All the samples (extracts, pure polyphenols, fluorescein and AAPH) were diluted in 75 mM phosphate buffer (pH 7.4). Thirty microliters of extracts (1 mg/mL) or phosphate buffer (blank) were mixed with 180 μL of fluorescein solution (117 nM final concentration) and incubated for 5 min at 37 °C. A volume of 90 μL of AAPH solution (40 mM final concentration) were added and fluorescence was immediately monitored using 485 nm excitation and 520 nm emission wavelengths at 1 min intervals for 70 min. The antioxidant capacities of extracts or purified molecules were expressed as mg of Trolox equivalent per g of extract (mg TE/g) or mmol equivalent Trolox per g of polyphenols (mmol TE/g P), respectively. All samples were analyzed in quadruplicate and at least in three different experiments.

### Cell culture and MTT assay of EtOAc fractions

Pheochromocytoma-derived PC12 cells (ATCC, Manassas, VA) were maintained routinely in DMEM-Glutamax supplemented with 15% horse serum, 2.5% fetal bovine serum and 1% penicillin/streptomycin antibiotics at 37 °C in a humidified atmosphere of 5% CO_2_/50% air. Cells were plated at a density of 30,000 cells per well on 96-well plates and incubated at 37 °C for 24 h. Then, the cells were treated with 5 μM of Aβ_(25 − 35)_, with or without extracts (25, 50, 100, 200 and 300 μM) or pure molecules (10, 25, and 50 μM) in a serum-free culture medium. After 24 h of incubation, cell viability was determined by the conventional MTT reduction assay. Cells were treated with MTT solution (0.5 mg/mL) for 3 h at 37 °C. The dark blue formazan crystals formed in viable cells were solubilized with DMSO for 0.5 h. The absorbance was measured at 595 nm with a microplate reader (Dynex, Carnegie, PA). Results were expressed as the percentage of MTT reduction in relation to the absorbance of control cells at 100%. All data represent the average of four tests.

### Liquid chromatography-mass spectrometry

Lyophilized EtOAc fractions were dissolved in 50% methanol and chromatographed using an HPLC-MS system. The chromatography apparatus was an Agilent 1200 from Agilent Technologies (Santa Clara, CA). The EtOAc fractions were analyzed at 25 °C with a 250 × 4 mm i.d., 5 μm, Prontosil 120-5-C18-AQ reverse phase column, Bischoff (Leonberg, Germany). Water, 0.1% HCOOH (solvent A) and acetonitrile 0.1% HCOOH (solvent B) were used as mobile phases. The gradient elution program was as follows (v/v): 0 min 1% B, 0.4 min 1% B, 2 min 10% B, 6 min 35% B, 7 min 50% B, 8.8 min 70% B, 10.8 min 92% B, 11 min 100% B and 12 min 100% B, followed by 10 min for re-equilibration. The optimum values of the ESI-MS parameters were: capillary voltage – 4.7 kv; drying gaz temperature 350 °C; drying gas flow 10 L/min; nebulizing gas pressure 35 psi. LC/MS accurate mass spectra were recorded across the range 150–2000 *m/z*. The detection wavelengths were set at 280 and 360 nm. LC-ESI-MS analyses were carried out in the negative ion mode. This HPLC was coupled to an Esquire 3000 + ion trap mass spectrometer using an ESI source from Bruker Daltonics (Billerica, MA).

### Preparative HPLC

In order to increase our production capacity of pure compounds, we used preparative HPLC Varian chain consisting of two pumps (model 218 with heads of 50 mL min^−1^ maximum flow rate) and a dual wavelength detector UV-visible wave (model 325). The flow rate was 18 mL min^−1^ with a C_18_ reverse phase column Bischoff Ultrasep Eurobond (5 μm, 20 mm diameter ×250 mm) protected by a C_18_ guard column (20 mm diameter ×50 mm). The solvents used are mixture of acetonitrile (ACN) and acidified water, the elution of phenolic compounds occurs more polar to less polar, driven by a mobile phase increasingly non polar. A stationary phase C_18_ reverse phase column and mobile phase solvent mixture in gradient fashion were used: solvent A (H_2_O/0.1% TFA) and solvent B (ACN/0.1% TFA). Throughout the chromatography, the concentration of trifluoroacetic acid (TFA) remains constant and provides good resolution of peaks. The gradient is according to the following order: 99–1% B (0–4 min), 99–1% B (4–12.8 min), 90% B (12.8–13 min), 10–100% B (13–55 min), 100% B (55–59 min). Injections of 3 mL/min to solutions of about 100 mg dry extract were dissolved in 1 mL of 50% MeOH.

### Statistical analysis

All samples were analyzed at least in triplicates. Data are expressed as means ± standard error mean (SEM). Differences were evaluated by one-way analysis of variance (ANOVA) completed by Tukey’s test. Differences were considered statistically significant at *p* < 0.05. Alternatively, the results were analyzed by GraphPad Prism 5.03 for Windows (GraphPad Software, San Diego, CA).

## Results and discussion

### Phenolic content *of F. pulverulenta*

The obtained fractions (crude extract, hexane, dichloromethane, ethyl acetate, butanol and water) from *F. pulverulenta* shoot and root were analyzed for their total phenolic contents (TPC). Results depicted in Table 1 show that the highest recovery of TPC was observed for extraction with EtOAc reaching up to 383 and 374 mg GAE/g in aerial part and roots, respectively. In both plant parts, the TPC was found to be in the order EtOAc > BuOH > methanol > water > dichloromethane > hexane, suggesting that polar solvents were more efficient in extracting phenolics from *F. pulverulenta*. However, given the low polarity of EtOAc (polarity index 4.4) when compared with butanol, methanol (5.1) or water (polarity index 9), it seems logical to suppose that the highest recovery of TPC by using EtOAc was presumably due to its high molecular weight (88 g/mol) which enables it to easily extract about the same molecular weight following the concept ‘like dissolve like’. At this point, it can be expected that most of the phenolic compounds were of catechin- or epicatechin-type compounds. Support to this assumption is given by Row and Jin (2006) who reported that catechin-type phenolics are better extracted with EtOAc.

**Table 1.** Total phenolic content (TPC) and antioxidant capacities of *F. pulverulenta* aerial part and roots fractions.

	Extract/fraction	TPC (mg of GAE/g)	DPPH (mg of TE/g)	ABTS (mg of TE/g)	ORAC (mg of TE/g)	MCA (mg of EDTA/g)
Aerial part	Crude extract	286 ± 10^c^	378 ± 42^c^	216 ± 33^d^	495 ± 43^c^	44 ± 3^b^
	Hexane fraction	71 ± 5^f^	88 ± 17^e^	165 ± 27^d^	196 ± 30^e^	25 ± 2^d^
	Dichloromethane	115 ± 7^e^	77 ± 10^e^	220 ± 22^d^	617 ± 58^b^	21 ± 2^d^
	Ethyl acetate	383 ± 10^a^	586 ± 31^b^	1453 ± 63^a^	821 ± 34^a^	37 ± 6^c^
	Butanol	319 ± 6^b^	698 ± 73^a^	1110 ± 67^b^	496 ± 38^c^	44 ± 4^b^
	Water	210 ± 9^d^	193 ± 51^d^	626 ± 35^c^	374 ± 51^d^	62 ± 3^a^
Root	Crude extract	202 ± 13^c^	540 ± 50^b^	230 ± 45^d^	398 ± 33^d^	15 ± 1^b^
	Hexane fraction	25 ± 3^f^	30 ± 10^d^	156 ± 10^d^	52 ± 17^f^	12 ± 1^c^
	Dichloromethane	105 ± 8^e^	45 ± 10^d^	230 ± 25^d^	501 ± 50^c^	12 ± 1^c^
	Ethyl acetate	374 ± 15^a^	750 ± 11^a^	1319 ± 76^a^	1054 ± 54^a^	23 ± 2^a^
	Butanol	289 ± 7^b^	529 ± 116^b^	1242 ± 54^b^	646 ± 32^b^	23 ± 2^a^
	Water	182 ± 6^d^	204 ± 66^c^	754 ± 41^c^	311 ± 23^e^	15 ± 1^b^

^a–e^Significant difference at *p* < 0.05 by Tukey’s test.

### Antioxidant activity of shoot and root fractions

Results depicted that EtOAc and BuOH fractions of *F. pulverulenta* exhibited the higher scavenging effects (Table 1). Moreover, root fraction exhibits better performance than the shoot fraction against DPPH and ABTS radicals. These results suggest that antioxidative phytochemicals are abundantly present in *F. pulverulenta*, particularly in EtOAc and BuOH fractions. These data are in agreement with the previous study of Saada et al. (2014), which showed that EtOAc fraction of the halophyte *Retama raetam* (Fabaceae) exhibits the highest total phenolic compounds and antioxidant activity. Desire et al. (2010) also found that the EtOAc fraction of *Mascarenhasia arborescens* had better antiradical capacity than the other fractions like hexane, water and methanol. Accordingly, the superiority of the EtOAc fraction is probably associated to its polarity which allows the accumulation of a variety of antioxidant compounds.

Measuring the ability of oxygen radical absorbance (ORAC) was widely used in the field of antioxidants and oxidative stress. The antioxidative potential of different fractions evaluated by ORAC indicated that EtOAc fraction is the most active (821 and 1054 mg TE/g for aerial parts and root, respectively). But, hexane fractions showed very low peroxyl radical scavenging activity, which was around 20 times lesser than EtOAc fraction (Table 1). In this context, Surget et al. (2015) observed that EtOAc fraction of the halophyte *Salicornia amosissima* showed high ORAC activity. It appears that the ORAC values in the present study were comparable to or even higher than those reported by Silva et al. (2007), where found ORAC values ranged from 6.7 to 1396.4 μmol of Trolox equiv/g in 15 medicinal Amazonian plants. These results support previous studies on antioxidant tests, since EtOAc fractions usually present the highest TPC and antioxidant capacities (Dhingra et al. 2014; Saada et al. 2014; Surget et al. 2015).

The metal chelating activities of *F. pulverulenta* fractions were monitored in order to evaluate the ability to inhibit interactions between metals and lipids. Water fraction exhibited the strongest chelating capacity as compared with the other fractions of aerial parts (62 mg EDTA/g) (Table 1). In roots, EtOAc and butanol fractions had the most important chelating activity (23 mg EDTA/g). In addition, shoot iron chelating activities were significantly higher than the root fraction. The results were in agreement with Dhingra et al. (2014) on the *Prunus persica* fruits, these authors showed that EtOAc and *n*-butanol fractions possess the higher Fe^2+^-chelating activity.

Due to the high TPC and the important antioxidant capacities of EtOAc fraction, the identification of the main bioactive compounds by LC-DAD-ESI-MS and the purification by preparative HPLC of major compounds of EtOAc shoot fraction were performed. The protective effect of these fractions and purified compounds against Aβ-induced toxicity in the PC12 cell line was further investigated.

### LC-DAD-ESI-MS screening

**Figure 1.**
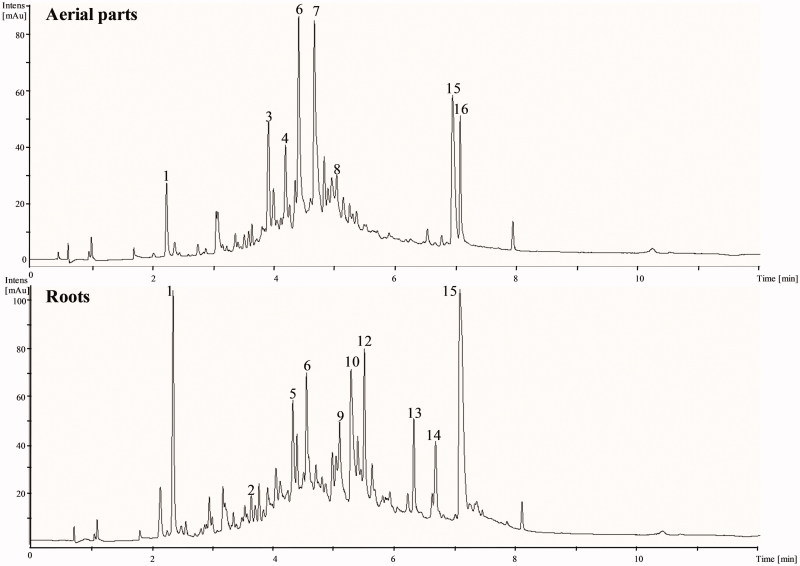
LC-DAD-ESI-MS base peak chromatograms in negative ion mode and UV at 280 nm for the ethyl acetate fraction of aerial parts and roots of *F. pulverulenta*.

Phenolic composition analysis of *F. pulverulenta* EtOAc shoot and root fractions has been carried out using LC-DAD-ESI-MS at a negative mode (Table 2). For the first time, the methodology used in this work, allowed us to identify nine new phenolic compounds (**1**, **2**, **3**, **4**, **6**, **7**, **8**, **10** and **11**) which have not been reported so far in this species (Figure 1). The compounds detected were characterized by means of MS data, together with the interpretation of the observed MS-MS spectra in comparison with those found in the literature public databases.

**Table 2.** Phenolic compounds detected in ethyl acetate fraction by LC-DAD-ESI-MS from aerial parts and roots of *F. pulverulenta* in negative mode.

Compound No	*R_*t*_* (min)	*λ*_max_(nm)	[M − H]^−^	Fragments	Organ	Compound
**1**	2.3	270-275	169	125	AP, R	Gallic acid
**2**	3.6	260	577	407-425-451-289	R	Procyanidin dimer 1
**3**	4	280	289	245-179-205	AP	Catechin
**4**	4.3	275	577	407-425-451-289	AP	Procyanidin dimer 2
**5**	4.3	275	551	291-352-415-465-529	R	ND
**6**	4.5	275	635	483-465	AP, R	Tri-galloyl hexoside
**7**	4.8	275	591	301-255-359-407-439-465-487-529	AP	Quercetin galloyl glucoside
**8**	5.1	270	577	407-425-451-289	AP	Procyanidin dimer 3
**9**	5.1	280	619	245-289-407-425	R	ND
**10**	5.3	280/370	301	301	R	Quercetin
**11**	5.3	285/360	477	301-151-179	AP	Quercetin hexoside
**12**	5.5	290/320	435	168-315-345	R	ND
**13**	6.3	280/365	551	343-491	R	ND
**14**	6.7	290/320	685	365-458-488-514-541-617-649-667	R	ND
**15**	7.1	290/360	423	343-80	AP, R	Flavonoid-sulfated isomer
**16**	7.2	290/310	423	343-80	AP	Flavonoid-sulfated isomer

Compound **1** at *m/z* 169 corresponds to gallic acid. It was identified with fragmentation ion at *m/z* 125 ([M − H-44]^−^ corresponding to the loss of carboxylate). Analysis revealed the presence of three isomers of procyanidin dimers (compounds **2**, **4** and **8**) in the ESI- mode with ions at *m/z* 577. It was identified with the same fragmentation ions at *m/z* 451, 425 (corresponding to the neutral losses of galloyl *m/z* 152), 407 (loss of gallic acid *m/z* 170) and 289 (Kabran et al. 2014).

Compound **3** (Rt 4 min) with the precursor ion at *m/z* 289 was assigned as catechin. MS/MS spectral data showed product ions at *m/z* 245, 179 and 205 (Kabran et al. 2014). Compound **6** was identified as trigalloyl hexoside (Rt 4.5 min) with precursor ions at *m/z* 483, corresponding to the loss of galloyl at *m/z* 465 (loss of gallic acid at *m/z* 170) (Abu-Reidah et al. 2015).

Compound **7** (Rt 4.8 mn; *λ*_max_ = 275) is proposed as quercetin galloyl glucoside, yielded a prominent fragment ion at *m/z* 301, [M–290–H], assigned to interflavanic bond cleavage following the quinone-methide mechanism (quercetin), *m/z* 255 [M − H_2_O-pentose moiety], *m/z* 439 [M–152–H] corresponding to the loss of the galloyl fragment (C_8_H_8_O_3_) from retro-Diels-Alder (RDA) cleavage; *m/z* 465 [M–126–H], to the loss of the fragment (C_6_H_4_O_2_) from RDA (Sannomiya et al. 2005).

Compound **10** at *m/z* 301 corresponds to quercetin and compound **11** had the [M − H]^−^ ion at *m/z* 477, which yielded the fragment ions at *m/z* 301 ([M − H-162]^−^ corresponding to the loss of one galactose or glucose unit), 179 and 151, identified as quercetin hexoside (Kabran et al. 2014).

Seven compounds (**5**, **9**, **12**, **13**, **14**, **15** and **16**) eluting respectively at 4.3, 5.1, 5.5, 6.3, 6.7, 7.1 and 7.2 remain unidentified. No data corresponding to their mass spectra in the literature and databases are reported. Among them, compounds **15** and **16** matched exactly with the accurate mass at *m/z* 423 and same fragment ions *m/z* 343 and *m/z* 80 corresponding to a loss of one sulfate and UV-Vis spectrum shape with a *λ*_max_ at 290 and 360 nm attributed to two isomer sulfated flavonoids. Isolation and NMR identification might be required to identify these compounds.

### Neuroprotective activity of EtOAc fractions

**Figure 2.**
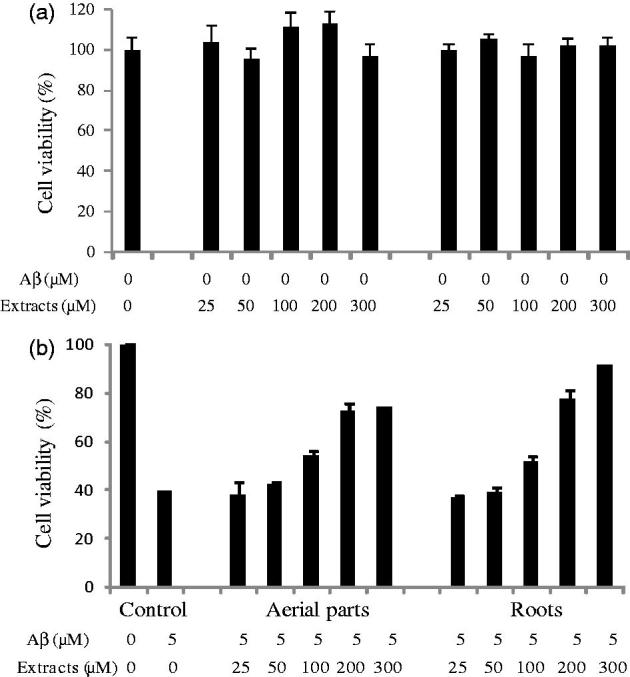
Cytotoxic activity (a) and neuroprotective activity on Aβ-induced toxicity in PC12 cell line (b) of aerial part and root EtOAc fraction. The experiment was repeated three times.

The cytotoxic potential of each phenolic fraction on PC12 cells was measured with the MTT assay. EtOAc fractions (*F. pulverulenta* shoot and root) were not cytotoxic to concentration up to 300 μg/mL (Figure 2(a)). As shown in Figure 2(b), treatment of PC12 cells with 5 μM Aβ_25–35_ reduced cell viability about 40% of control. Induction of cytotoxicity by Aβ_25–35_ at 5 μM was then used for all subsequent experiments to evaluate the protective effect of the species. Both EtOAc fractions from shoot and root exhibited cytoprotective activities against Aβ-induced toxicity. The extracts increased cell viability in a dose-dependent manner. Incubation of PC12 cells with 100 μg/mL of fractions significantly prevented the cytotoxic effect of Aβ_25–35_ at levels close to 57%. Aβ-induced cytotoxicity was prevented at levels close to 80% at 200 μg/mL. The root fraction at the highest concentration tested (300 μg/mL) completely reversed the toxic effect of Aβ_25–35_. This cytoprotective activity is mainly correlated to the nature of phenolic compounds in these fractions. In this context, several reports have demonstrated that Lotus seedpod proanthocyanidins act as anti-aging agents for protection against memory deficits (Xiao et al. 2015).

### ORAC assay and neuroprotective effect of purified compounds

With regard to the discussion on phytochemicals responsible for antioxidant and cytoprotective capacities, it could be assumed that these properties were attributed to high content of phenolics such as gallic acid, quercetin, quercetin galloyl glucoside, catechin, trigalloyl hexoside and procyanidine dimers. Thus, the newly isolated phenolics from *F. pulverulenta* EtOAc fractions were tested for their ORAC and neuroprotective activities. Results presented in Table 3 show that quercetin had the highest ORAC values (33.558 mmol TE/g polyphenols), followed by catechin (21.917 mmol TE/g polyphenols) and gallic acid (14.176 mmol TE/g polyphenols). Trigalloyl hexoside and quercetin galloyl glucoside showed very low peroxyl radical scavenging activity, which was around 11- and 28-fold weaker than quercetin. Moreover, this study displayed quercetin ORAC values comparable withthat reported by Dàvalos et al. (2004).

**Table 3.** Antioxidant capacity (ORAC) of purified molecules of the ethyl acetate fraction.

Pure molecule	ORAC (mmol TE/g polyphenols)
Gallic acid	14.176
Catechin	21.917
Procyanidin dimer	4.71
Trigalloyl hexoside	2.99
Quercetin galloyl glucoside	1.2
Flavonoid sulphate	ND
Quercetin	33.558

The results are expressed in mmol Trolox equivalent/g polyphenols.

**Figure 3.**
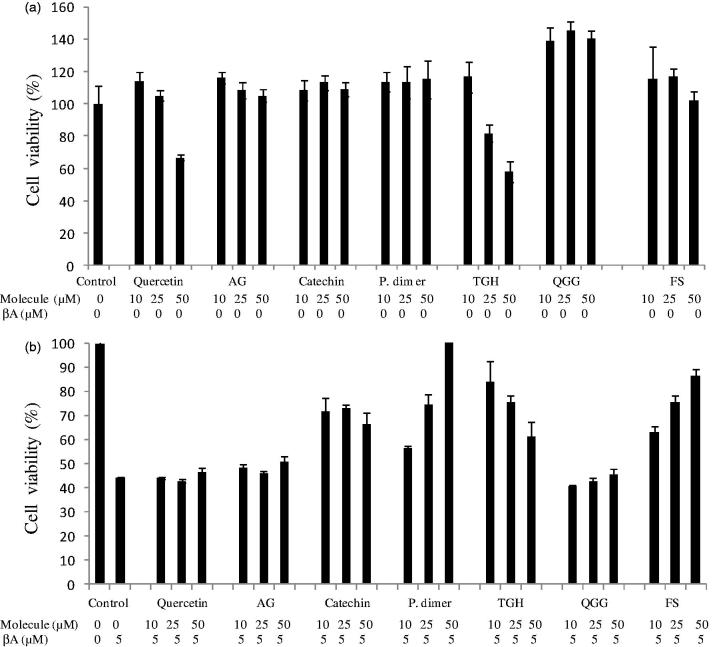
Cytotoxic activity (a) and neuroprotective activity on Aβ-induced toxicity in PC12 cell line (b) of purified molecules of the ethyl acetate fraction. GA: gallic acid; P dimer: procyanidin dimer; TGH: trigalloyl hexoside; QGG: quercetin galloyl glucoside; FS: flavonoid sulfated.

Concerning MTT test, like EtOAc fractions, main purified molecules did not significantly affect cell viability, with the maximum concentration of 50 μM (Figure 3(a)). Only quercitin (at 50 μM) and trigalloyl hexoside (at 25 and 50 μM) were cytotoxic on PC12 cells. Major purified phenolics prevent the aggregation of Aβ (Figure 3(b)). Among them, procyanidin dimer exhibited an extremely efficient inhibition of Aβ-induced cell death in PC12 cells, followed by flavonoid sulfate, in a dose-dependent manner. Aβ-induced cytotoxicity was prevented at levels close to 100% and 85%, respectively, at 50 μM. Trigalloyl hexoside prevented also significantly the cytotoxic effect of Aβ_25–35_ to 86% at the lowest concentration (10 μM) and decreased at higher concentrations to 60% (50 μM). This decrease is due to the cytotoxic effect of trigalloyl hexoside up to 10 μM on PC12 cells. Also, quercetin, quercetin galloyl glucoside and catechin are not efficient neuroprotective agents. In fact, one of the major properties of polyphenols is the important interaction with peptides and proteins, particularly in the AD (Henry-Vitrac et al. 2010). Several studies indicate that polyphenols present in high amounts in natural products could play a preventive role in the incidence of age-related neurological disorders (Basli et al. 2004). These findings have been supported by epidemiological studies and confirmed by *in vitro* and *in vivo* studies (Ono et al. 2003; Bastianetto et al. 2006). The ability of EtOAc fractionation in phenolics can explain their capacity to reduce the cell death caused by Aβ.

## Conclusion

To our knowledge, this is the first report characterizing the phenolic profiles, antioxidant and neuroprotective capacities of *F. pulverulenta* species. This study showed that extraction solvents had a significant impact on the phenolic contents and against various oxidative systems and metal-chelating activity under *in vitro* condition. Overall, EtOAc contained the highest level of antioxidant activities. Among purified bioactive molecules in this fraction, procyanidin dimer, trigalloyl hexoside and sulfate flavonoid revealed potent neuroprotective capacity which might constitute a novel source of neuroprotective compounds.
